# Supplementary material for: Concentrations of potentially toxic metals and trace elements in pregnant women and association with birth outcomes: A cross-sectional study in Malawi
Source: PLOS Glob Public Health. 2024 Nov 14;4(11):e0003521. doi: 10.1371/journal.pgph.0003521 (PMC11563382; doi:10.1371/journal.pgph.0003521)
Supplement: Attachment — Submitted filename: Response To Reviewers.pdf [file pgph.0003521.s002.pdf]

Mphatso Mwapasa
Department of Public Health and Nursing
Faculty of Medicine and Health Sciences
Norwegian University of Science and Technology
**TRONDHEIM**
Norway
E-mail: mphomwapasa@gmail.com

**24th September 2024**.

The Editor
Plos Global Public Health

Dear Editor,

Concentrations of potentially toxic metals and trace elements in pregnant women and association with birth outcomes: A cross-sectional study in Malawi." – Manuscript resubmission for peer review and publication

**Journal Requirements:**

1. Please include a complete copy of PLOS' questionnaire on inclusivity in global research in your revised manuscript.

**Response:** *PLOS' questionnaire on inclusivity in global research has now been included in my submission as a separate file labeled "Inclusivity-in-global-research-questionnaire"*

2. We have amended your Competing Interest statement to comply with journal style. We kindly ask that you double check the statement and let us know if anything is incorrect.

**Response:** *The changes are correct.*

3. We do not publish any copyright or trademark symbols that usually accompany proprietary names, eg (R), (C), or TM (e.g. next to drug or reagent names). Please remove all instances of trademark/copyright symbols throughout the text, including ® on page 6.

**Response:** ® *has been deleted on page 6 of the manuscript. Furthermore, the authors have skimmed through the manuscript to make sure that there no copyright or trademark symbols (Page 6, line 151)*

4. In the online submission form, you indicated that "Data would be made available on request from the corresponding author." All PLOS journals now require all data underlying the findings described in their manuscript to be freely available to other researchers, either

a. In a public repository,
b. Within the manuscript itself, or
c. Uploaded as supplementary information.

**Response:** *Dataset for this study can be accessed on the public repository (github repository) by following the link below:*
*https://github.com/MphatsoMwapasa/Toxic-essential-Metals-Data.git*

5. Figure 1: please (a) provide a direct link to the base layer of the map (i.e., the country or region border shape) and ensure this is also included in the figure legend; and (b) provide a link to the terms of use / license information for the base layer image or shapefile. We cannot publish proprietary or copyrighted maps (e.g. Google Maps, Mapquest) and the terms of use for your map base layer must be compatible with our CC-BY 4.0 license.

**Response:** *The figure has been replaced. The new map has been created the map in like R and the source of the basemap shapefile onto which data has been plotted has also been provided. (Page 5, line 109). The direct link to the base layer of the map has been provided in the figure legend and content on this site is licensed under a Creative Commons Attribution 4.0 (CC-BY 4.0 license) (Page 5, lines 110-111).*

**Comments to the Author**

Reviewer #1:
From my perspective, this article is notably interesting due to the type of population it studies, as it provides valuable information about a community that has been less explored in the scientific literature. It is particularly relevant to investigate how environmental exposures influence childhood growth and development in these less-studied areas. Delving deeper into this aspect could reveal specific patterns and effects that have not been identified in more widely investigated contexts.
However, I have identified several areas for improvement that could enhance the rigor and validity of the study. Firstly, there is a notable lack of review and application of the most current methodologies in environmental epidemiology. Advanced techniques such as Bayesian Kernel Machine Regression (BKMR), Weighted Quantile Sum Regression (WQSR), and gComputation are powerful tools for analyzing the potential effects of heavy metal exposure and its relationship with child health outcomes. These methodologies allow for a more robust and precise evaluation of multiple exposures and their interactive effects, significantly improving the quality of the analysis.
Additionally, in the sample analysis methodology section, the Limit of Detection (LOD) for each analyzed metal is not specified. The omission of this information is critical, as the LOD determines the sensitivity of the analysis and, consequently, the accuracy with which low concentrations of heavy metals can be detected. It is essential to report the percentage of samples below the LOD and to describe in detail the treatment of these data, as strategies for handling values below the LOD can significantly influence the results and their interpretation.
Furthermore, I have noticed that the discussion is organized into sections, which is not typical for studies of this type. Although a sectioned structure can offer greater clarity in

presenting results and conclusions, it can also fragment the discussion and hinder the integration of findings into a coherent narrative. I believe that a more fluid and continuous discussion could facilitate the understanding and impact of the presented results.
Incorporating these elements would not only strengthen the scientific validity of the study but also allow for greater comparability with similar studies, thus facilitating the integration of the findings into the broader body of knowledge on environmental epidemiology and child health.

**Response:** *In addition to Multivariable linear regression analysis to assess the effect of the maternal concentration of each potentially toxic metals and birth outcomes, we also used Weighted Quantile Sum Regression (WQSR) to analyze the combined effect of multiple exposure exposures, while considering their potential joint contribution to the birth outcome (Page 10, line 237 - 245). In this regard, figure 3-footer has also been replaced to include results from the additional analysis (Page 17, line 384 - 386). Furthermore, the discussion of results from the WQSR analysis have also been added to the discussion section (Page 26, lines 564 - 566)*

*The authors believe that organizing the discussion into distinct sections, as done in this manuscript, enhances clarity and improves the overall readability for the audience. By structuring the content this way, complex ideas are broken down into manageable parts, allowing readers to follow the argument more easily.*

*We acknowledge that the Limit of Detection (LOD) is reported for each metal analyzed in this study. However, the authors have used method detection limits (MDLs) as a proxy for LOD, given that both terms are often used interchangeably in the context of analytical sensitivity. While LOD typically refers to the smallest concentration of an analyte that can be reliably distinguished from background noise, MDLs represent a similar threshold based on the specific method employed in the analysis.*

**Additional revision:**

The word "metals" has been added in the title manuscript *(Page 1, line 1)*.

Your consideration over our resubmission will be greatly appreciated

Yours sincerely,

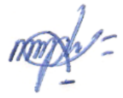

MPHATSO MWAPASA
